# A co-creation roadmap towards sustainable quality of care: A multi-method study

**DOI:** 10.1371/journal.pone.0269364

**Published:** 2022-06-30

**Authors:** Fien Claessens, Deborah Seys, Jonas Brouwers, Astrid Van Wilder, Anneke Jans, Eva Marie Castro, Luk Bruyneel, Dirk De Ridder, Kris Vanhaecht

**Affiliations:** 1 Leuven Institute for Healthcare Policy–Department of Public Health, KU Leuven–University of Leuven, Leuven, Flanders, Belgium; 2 Department of Orthopaedics, University Hospitals Leuven, Leuven, Flanders, Belgium; 3 Department of Quality Management, Sint-Trudo Ziekenhuis, Sint-Truiden, Flanders, Belgium; 4 Department of Quality Management, Regionaal Ziekenhuis Heilig Hart Tienen, Tienen, Flanders, Belgium; 5 Department of Quality Management, University Hospitals Leuven, Leuven, Flanders, Belgium; University of Salento, ITALY

## Abstract

**Objective:**

Hospitals demonstrated increased efforts into quality improvement over the past years. Their growing commitment to quality combined with a heterogeneity in perceptions among healthcare stakeholders cause concerns on the sustainable incorporation of quality into the daily workflow. Questions are raised on the drivers for a sustainable hospital quality policy. We aimed to identify drivers and incorporate them into a new, unique roadmap towards sustainable quality of care in hospitals.

**Design:**

A multi-method design guided by an eight-phase approach to develop a conceptual framework consists of multiple, iterative phases of data collection, synthesis and validation. Starting with a narrative review followed by a qualitative in-depth analysis and including feedback of national and international healthcare stakeholders.

**Setting:**

Hospitals.

**Results:**

The narrative review included 59 relevant papers focusing on quality improvement and the sustainability of these improved quality results. By integrating, synthesising and resynthesizing concepts during thematic and content analysis, the narrative review evolved to an integrated, co-creation roadmap. The Flanders Quality Model (FlaQuM) is presented as a driver diagram that features six primary drivers for a sustainable quality policy: (1) Quality Design and Planning, (2) Quality Control, (3) Quality Improvement, (4) Quality Leadership, (5) Quality Culture and (6) Quality Context. Six primary drivers are described in 19 building blocks (secondary drivers) and 104 evidence-based action fields.

**Conclusions:**

The framework suggests that a manageable number of drivers, building blocks and action fields may support the sustainable incorporation of quality into the daily workflow. Therefore, FlaQuM can serve as a useful roadmap for future sustainable quality policies in hospitals and for future empirical and theoretical work in sustainable quality management.

## Introduction

Twenty years ago, the US Institute of Medicine (IOM) defined healthcare quality and called for system changes to build a safer healthcare system [1]. During the past decade, important quality insights are offered into the complex work of healthcare as a dynamic entity constantly transforming to meet the needs of people for better health [2, 3]. Lachman and colleagues reflected about the relevance of IOM’s 20-year-old definition of quality and proposed a revised, multidimensional quality model including new domains, such as kinship, ecology and transparency. This model reflects the global change of healthcare quality management [4].

Healthcare organisations worldwide have been initiating quality improvements and building a foundation for quality by applying many theories, methodologies and interventions [5, 6]. In Flanders, Belgium, accreditation, public reporting and governmental inspection have been the main pillars for the development of hospital’s quality management system [7]. However, a recent review has shown that the current evidence about the impact of these pillars on patient processes and outcomes is scarce [8]. Moreover, hospitals’ increasing commitment to quality resulted in a heterogeneity in perceptions and attitudes towards quality initiatives among healthcare stakeholders [9, 10]. In the past year already ten Flemish hospitals announced their intention to leave hospital-wide accreditation [11], as many Danish and Dutch hospitals did some years ago [12, 13]. A growing “quality fatigue” is imminent in hospitals [14, 15]. Questions are rising about the sustainability of current initiatives [16, 17]. National and international hospital associations are looking for a future, sustainable quality management system [18, 19]. Once hospitals have taken the first steps to improve quality, it is important but challenging to sustain the gained quality results and ultimately achieve quality improvement as an integral part of the organisation culture [20–22].

However, evidence in the area of sustaining quality into the daily workflow of healthcare professionals is still lacking [22, 23]. First, there is no universal definition, conceptual consistency nor operational clarity for measuring sustainability [22, 24–26]. In literature only more general descriptions are found [23, 24, 27]. Second, most implementation studies do not report the success factors or essential activities for obtaining sustainability [27, 28]. Few studies explored influencing activities, which are mainly related to infrastructures [24–26, 29], human elements [24, 25, 29, 30], organisational and environmental support [24, 25, 29] and improvement initiatives [24–26, 29, 30]. Third, to the best of our knowledge, sustainability is only investigated as a minor part of the implementation process and not as a main pillar for quality management until now [22]. These three elements make it difficult to define broad key themes, hereinafter referred to as ‘drivers’, contributing to sustainable healthcare quality and to introduce them into real-world practice settings [24, 26]. Despite the current evidence [23–25, 29, 30], it remains unclear how to translate these results into a meaningful roadmap to incorporate quality into the daily workflow and culture from bedroom to boardroom.

In conclusion, drivers for a sustainable quality management system are essential in hospitals, but the lack of existing evidence show a real literature gap. The purpose of this study is to identify and describe different drivers to incorporate quality sustainably into the daily workflow. Furthermore, we aim to integrate these drivers into an evidence-based framework and roadmap for hospitals towards sustainable healthcare quality.

## Materials and methods

### Study design

A multi-method design was used, based on Jabareen’s eight-phase approach to develop an integrative framework [31]. This eight-phase approach have been used extensively in medical and health services research [32–36] and involves both a narrative review of literature and qualitative research. Our multi-method design contains 1) an in-depth analysis of a wide range of articles and reports and 2) seven group discussions with different healthcare stakeholders until consensus was reached.

### Data collection and analysis

In the first phase our objective was to identify drivers to incorporate quality sustainably into the daily workflow. Therefore, a narrative review was performed. Papers were retrieved in three ways (S1 Fig). First, we searched in MEDLINE and Google Scholar search engine for review articles published from January 2010 to October 2020. This date range was chosen in order to review recent advances and updated information in this particular field and to improve the efficiency and accuracy of the search. The main key words and MeSH terms were related to ‘framework’, ‘sustainability’ and ‘healthcare’. Second, we searched, based on advice of experts from the International Society for Quality in Health Care, online for internationally recognised (research) institutes in healthcare quality and included grey literature, like (white) papers or reports, published on their websites. These were included in the narrative review if relevant to the study. Third, (inter)national experts in healthcare quality policy recommended literature to complement the search results. All research articles and grey literature reports were purposively screened for selection criteria (Table 1) by one author (FC) and reviewed by two other authors (DS, KV). The reference lists of included papers were examined for potentially relevant literature not captured in the original search.

**Table 1:** Selection criteria.

Inclusion criteria:	• Peer-reviewed journal articles (secondary research: literature reviews) and grey literature reports • Written in English or Dutch • Published between January 2010 and October 2020 • Healthcare settings including hospitals, healthcare organisations and community health • Full text available via our institutions’ subscriptions or freely available on the Internet
Exclusion criteria:	• Written in languages other than English or Dutch • Other settings than the healthcare setting • If not relevant to the hospital context • Full text not available

In the second phase, extensive reading and categorising of the selected data for relevant concepts to be included in the framework involved a qualitative, in-depth thematic analysis with the NVivo12 software program. Thematic analysis in this framework development refers to the process of identifying and collating meaningful sections of the document text, such as describing the possible contribution of concepts to sustainable healthcare quality. Each article or report was screened for concepts by one author (FC) and discussed with two other authors (DS, KV). Based on these concepts, the first codes were constructed in NVivo12, which were adapted and restructured during the next phases according to new insights by rereading the literature.

During the third phase, content analysis was used to examine how patterns of concepts within and between documents emerge as broad key themes representing sustainable healthcare quality. With NVivo12 the most frequented terms were clustered–combining related terms such as ‘staff commitment and attitudes’, ‘empowerment’ or ‘engagement’–independently by the research team into these broad key themes, hereinafter referred to as ‘drivers’. Furthermore, by using the constant comparison method and thus extensive reading and rereading the literature more themes emerged and refined insights were created into the meaning of these drivers. The preliminary results were discussed at regular intervals by the research team.

In the fourth phase, primary drivers were refined by constructing key secondary drivers, hereinafter referred to as ‘building blocks’. The primary drivers and building blocks are categorised and organised according to their features as described in the included literature. By discussions within the research team, any discrepancies in categorisation were resolved and assessed in terms of underlying assumptions, interdependencies and relationships between concepts. This process was repeated by the researchers on a regular basis, individually and as a team, to increase the level of integration of drivers and building blocks. Moreover, the actionability of concepts for hospitals are kept in mind by developing action fields derived from the literature. A first visual representation was developed iteratively in a driver diagram with four columns. This easy-to-read visual display was chosen because it allows to add or eliminate drivers and building blocks identified during the validation phase [37].

Drivers and building blocks the research team has agreed on having similarities or big differences are aggregated or separated into new ones in the fifth phase.

During the sixth phase, the findings from phase 1–5 are synthesized into an integrated framework. As highlighted by Jabareen this phase is “iterative and includes repetitive synthesis and resynthesis until the researcher recognises a general theoretical framework that makes sense” [31]. Each building block and the incorporation into a graphical designed roadmap was discussed in detail with the research team.

To validate the content of the conceptual framework in the seventh phase, the graphical designed roadmap is presented to a Flemish healthcare stakeholder group (n = 33). This purposive stakeholder group is reflected by its disciplinary breadth with expert representation across a range of health areas: board members of hospitals (n = 12), policymakers (n = 6), representatives of patient associations (n = 3), representatives from the hospital umbrella organisation (n = 4) and scientists from different universities with experience in healthcare quality (n = 8). By combining stakeholders’ varying expertise into the further development of the roadmap, the graphical design is further refined to clearly display the relation and characteristics of the drivers, building blocks and action fields.

In the eighth phase, the roadmap is presented to hospital board members, quality steering groups and to various healthcare disciplines and clinicians in one small regional hospital and one large academic medical centre in Flanders, Belgium. The theoretical roadmap is rethought according to new insights and feedback from healthcare stakeholders working in a real-world setting.

## Results

### Building the quality roadmap

**Fig 1:**
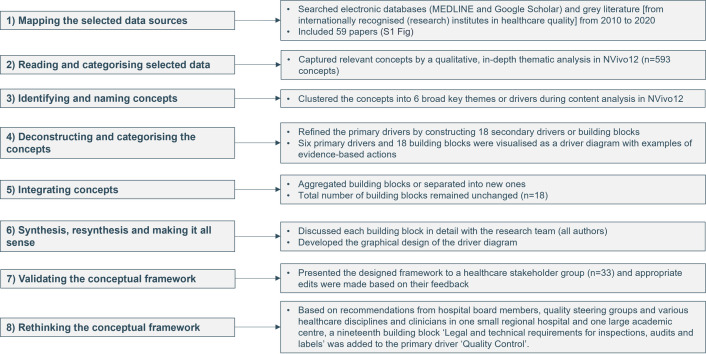
Eight-phase framework development approach.

**Fig 2:**
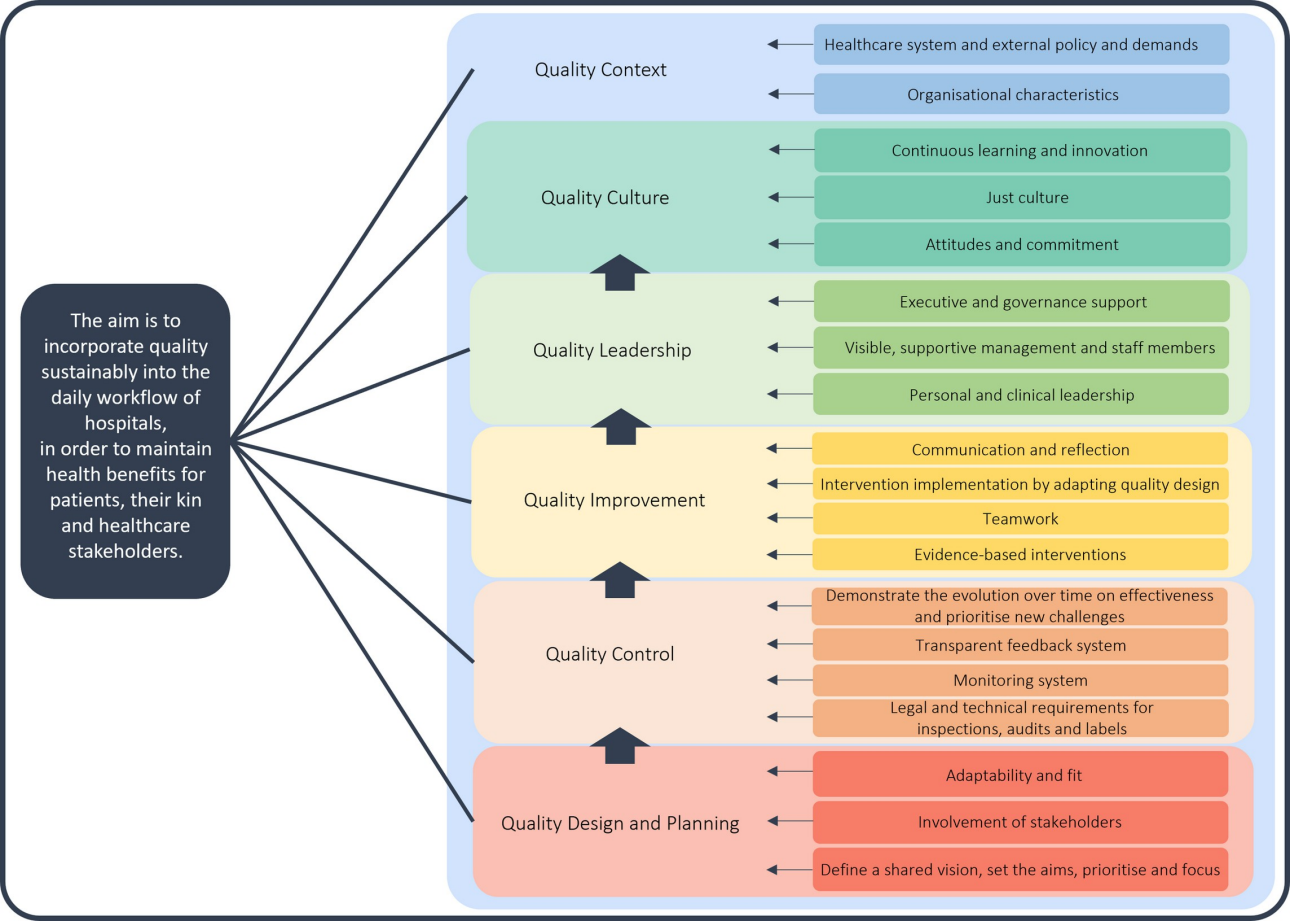
Integrated co-creation roadmap towards sustainable quality of care. Each colour represents a different driver. Each driver is linked to at least two building blocks. It is recommended to read this roadmap from the bottom to the top, starting with ‘Quality Design and Planning’. ‘Quality Context’ is visualised as an overarching driver.

The results of the eight-phase development approach are visualised in Fig 1. A total of 59 papers (28 research articles and 31 grey literature reports) fulfilled the selection criteria (i.e. describing a conceptual framework or model or mentioning concepts related to quality improvement and its sustainability) in the narrative review (S1 Table). During the thematic analysis of the included papers, 593 relevant concepts were captured. In the third and fourth phase, these concepts were clustered into primary drivers and building blocks and visualised as a driver diagram (Fig 2). This concept-mapping process included scientists from our research team with different experiences in healthcare (nurses, pharmacist, physicians, and experts in methodology and data). A driver diagram was constructed with the first column including primary drivers (n = 6); the second including building blocks related to primary drivers (n = 18); the third including change ideas in the form of evidence-based action fields per building block (n = 100); and the fourth presenting the references (n = 59) for each action field (S2 Table). Next, we integrated the drivers and building blocks. Finally, the results of all meetings with our research team, including the graphical design of the driver diagram as a roadmap) were discussed during a consensus meeting of this sixth phase. Thereafter, the roadmap was presented in the two last phases to a healthcare stakeholder group (n = 33) and hospital board members and clinicians from one small regional hospital and one large academic medical centre. Based on their recommendations, the roadmap was rethought by adding a nineteenth building block ‘Legal and technical requirements for inspections, audits and labels’ in primary driver ‘Quality Control’. By doing so, we created a hospital-wide roadmap, focusing on both care departments and technical departments. Furthermore, this new building block is made actionable by formulating four action fields.

### Drivers, building blocks and evidence-based action fields

The final result of the framework development approach is visualised in a driver diagram (Fig 2). This framework includes six main drivers at the core of the diagram: (1) Quality Design and Planning, (2) Quality Control, (3) Quality Improvement, (4) Quality Leadership, (5) Quality Culture and (6) Quality Context. The order between the drivers is visualised as a roadmap, starting with the drivers ‘Quality Design and Planning’, ‘Quality Control’ and ‘Quality Improvement’. The next driver is ‘Quality Culture’. To reach this culture throughout the organisation, ‘Quality Leadership’ at every hospital level is needed. The roadmap ends with taking the ‘Quality Context’ of the real-world setting into account. The drivers are feeding into each other and related to 19 building blocks. These are described in detail below. To make building blocks actionable for organisations, 104 evidence-based action fields are formulated (S2 Table).

#### Driver 1: Quality design and planning

The first driver contains three building blocks: (1) ‘Define a shared vision, set the aims, prioritise and focus’, (2) ‘Involvement of stakeholders’ and (3) ‘Adaptability and fit’. The first building block is the starting point of the co-creation roadmap. In this building block, the focus should be on creating a shared vision from a multidimensional perspective reflecting in everything the organisation does [4]. To define the shared vision, organisations need to create a ‘people’-matter mindset through involving stakeholders from the inception towards sustainability. By involving stakeholders, their perspectives, experiences, interests and needs are understood and competing demands are made transparent. Additionally, it is important that the shared vision and aims are not only adapted in the language, culture and structure of the organisation but also fits with internal and external demands and priorities.

#### Driver 2: Quality control

With keeping in mind the shared vision and aims established in driver one, the second driver focuses on controlling the quality of organisations. This driver consists of four building blocks: (1) ‘Legal and technical requirements for inspections, audits and labels’, (2) ‘Monitoring system’, (3) ‘Transparent feedback system’ and (4) ‘Demonstrate the evolution over time on effectiveness and prioritise new challenges’. Legal and technical requirements and an up-to-date overview of these requirements are the basis to drive quality control in healthcare organisations. Within the monitoring system, a mix between different kind of indicators and a balance between soft and hard metrics are the focus. This system needs to ensure benchmarking, focus on variation and longitudinal follow-up of healthcare quality. Results from the monitoring system will be transparent to all internal and external stakeholders through implementing a real-time feedback system. Thus, the level-of-detail for data (aggregated or individual data) is defined and the target audience understands the data flow. By focusing on trends, the evolution over time on effectiveness will be demonstrated and new quality challenges will be prioritised.

#### Driver 3: Quality improvement

After planning for quality and further defining quality measures, the focus should be on how to improve quality in order to reach benefits for patients, their kin and healthcare stakeholders. The driver ‘Quality Improvement’ consists of four building blocks: (1) ‘Evidence-based interventions’, (2) ‘Teamwork’, (3) ‘Intervention implementation by adapting quality design’ and (4) ‘Communication and reflection’. To improve findings of the monitoring system, evidence-based interventions can be developed by combining research, practice and experiences of patients, kin and staff. Important during this intervention development is to focus on identifying symptoms and causes of poor quality within current organisation processes. Evidence-based interventions need to be implemented by means of multidisciplinary teamwork, including team members with different skills, experiences, knowledge and viewpoints. Given the complexity of healthcare work processes, these teams can further adapt the quality design by intervention implementation with respect to the science of human factors engineering. The why, the content and the change methodology of the new design should be clearly communicated to involved stakeholders. They need to reflect about this new design to understand the relation between the intervention, the implementation method and the outcomes for patients, their kin and providers.

#### Driver 4: Quality leadership

In order to achieve successful implementation and sustainability of the first three drivers, leadership in quality is needed. This quality leadership is defined on three different organisational levels, which are described with three building blocks: (1) ‘Personal and clinical leadership’, (2) ‘Visible, supportive management and staff members’ and (3) ‘Executive and governance support’. Every healthcare provider ensures to work as a purposeful, committed, inspirational and critical leader who tries to understand for example the needs of patients, their kin and colleagues. In practice, they participate to codesign quality initiatives and actively support the organisational goals, the monitoring and feedback system and the implementation of quality initiatives. Ongoing support for these clinical and personal leaders through the management and staff members is ensured by focusing on ‘a systems view’ and showing dignity and respect to all stakeholders. Executives and boards further ensure that quality is the strategic centre of everything the organisation does. Like all staff, they demonstrate active contribution, involvement and commitment to quality design and planning, quality control and quality improvement.

#### Driver 5: Quality culture

Leadership can create and reinforce the organisational culture in quality, which forms the fifth driver ‘Quality Culture’. Three building blocks are incorporated in this driver: (1) ‘Attitudes and commitment’, (2) ‘Just culture’ and (3) ‘Continuous learning and innovation’. Probably the most common aphorism related to managing people or organisations is that “culture eats strategy for breakfast”. In view of this, we assume that everybody lives the core values of quality (e.g. partnership and coproduction, dignity and respect, holistic care, kindness with compassion). Everybody needs to be motivated, engaged, ready for change and beliefs in the quality design and planning, quality control and quality improvement. Moreover, this means that all staff take ownership and show accountability for their relations with every patient, their kin, colleagues and the organisation. Balancing between accountability and support at every organisational level should be supportive for ‘a just culture’, defined as treating individuals fairly and justly “when things go wrong”. It is important that all patients, kin and staff experience this blame free environment with trust and inclusion, to create a continuous learning culture. A learning system embeds the ability to continuously learn from errors/near misses as well from positive outcomes. This can be the basis for an embedded quality and improvement culture where all staff get the opportunity to learn, from safety-I to safety-II.

#### Driver 6: Quality context

The last driver, ‘Quality Context’, is an overarching driver, that has an influence on the other five drivers. This driver contains two building blocks: (1) ‘Organisational characteristics’ and (2) ‘Healthcare system and external policy and demands’. First, available financial and technical resources, an unambiguous structure from boardroom to bedroom, a competency framework, a capacity and building system and collaboration with external partners are examples of organisational characteristics that supports in the sustainability of healthcare quality. Second, the legislation, ethical and governmental commitment, ensuring financial incentives, the supporting role of external governmental and non-governmental bodies and the external societal demands are healthcare systems’ characteristics and external policy and demands. Healthcare organisations cannot change these external characteristics of the system themselves.

## Discussion

This article describes the development of a Flanders Quality Model (FlaQuM) as a new roadmap towards a holistic, integrated approach to sustainable quality management. By reconciling integrative research including a qualitative in-depth analysis of our narrative review and input from international and national healthcare experts, we built a roadmap including six drivers, 19 building blocks and 104 evidence-based actions supporting sustainable quality management in hospitals. The development of the framework was non-sequential and iterative in nature, by moving between data collection and analysis, evolving in an eight-phase approach. The qualitative method of data collection created the opportunity for the Flemish healthcare stakeholder group to include additional items that were not addressed in the narrative review. This integrative research with a multi-method design fostered to integrate quality concepts into one roadmap, while putting attention to the complexity of sustainability and its holistic approach. These strong empirical foundations underpinning the co-creation roadmap enhance the theoretical validity and clinical relevance, with several possible evidence-based actions derived from our included literature. Quality models which are co-created with stakeholders and are able to sustain in the workflow are more likely to deliver health benefits for patients and healthcare stakeholders [38, 39]. Furthermore, by letting quality grow from bottom-up, organisations can regain their diminished commitment to quality which was due to the imposed and bureaucratic feeling of accreditation systems [40]. By focusing on involving and creating value for all stakeholders, from boardroom to bedroom and from healthcare stakeholders to patients, there is the opportunity for patients to take an active role in healthcare quality [41, 42]. The roadmap shows hospitals the way to a sustainable healthcare quality system through a step-by-step approach focusing on the organisation’s priorities and how this can be built up in the organisation’s context. In terms of sustainability, all primary drivers are equally important, but we note that most attention at the start of sustainability may go to the first three primary drivers. These three drivers are similar to traditional concepts derived from Juran’s Trilogy [43]. Hospitals starting the roadmap with the driver ‘Quality Design and Planning’, can keep in mind the multidimensional quality model reflected by Lachman and colleagues [4]. The six drivers all feature prominently in existing literature of quality management systems, for example white papers and reports from 15 internationally recognised (research) institutes in quality and safety, for example the Agency for Healthcare Research and Quality [44], the World Health Organisation [45] and the Organisation for Economic Co-operation and Development are included in the literature review [46]. Current research provides only a partial picture of quality management, for example research about a single driver or building block [47–50]. This roadmap is, to the best of our knowledge, uniquely poised to promote sustainable quality incorporation into the daily workflow as a holistic, integrated approach for hospital quality management. The benefit of the roadmap is that organisations can start at any position on the roadmap and any moment in time. This can happen, for example, by defining priorities for next year.

### Strengths and limitations of the study

This study is strengthened by its wide scope achieved by means of the narrative review that explored publications, grey literature in the wider context of healthcare and other key references by applying a snowball approach [51]. This reflects the broad field of quality models and management systems in place. To derive drivers, building blocks and action fields towards sustainable healthcare quality from literature, a multi-method design was guided by Jabareen’s integrative research approach, which has proven its methodological value to obtain content validity in previous healthcare research [32–36]. By including feedback from multidisciplinary healthcare stakeholders, including clinicians, managers, policymakers and patient representatives, to refine the framework, its clinical and managerial relevance across disciplines is ensured [52]. While the roadmap is presented by the simple visualisation of a driver diagram, it encapsulates considerable complexity and requires substantial effort to implement the features into practice [34]. To support this implementation in a pragmatic and tangible way for hospitals, the roadmap is refined with 104 evidence-based action fields.

Despite these strengths, there are important limitations that need to be highlighted. First, we used narrative review methods instead of a systematic search to collect literature. This is a recognisable methodological limitation; some papers may have been overlooked. However, we attempted to address this potential limitation by consulting international experts in healthcare quality. The purpose was to collect concepts that have been used to sustainable quality and the conceptually grounded method described by Jabareen better aligned with that purpose. Second, the roadmap should be further analysed to understand the international, organisational and cultural differences. However, by including peer-reviewed papers and reports from international institutes in healthcare quality, this roadmap could support all types of hospitals experiencing similar challenges with respect to their specific context.

### Practice implications and future research

This co-creation framework provides a theoretical roadmap to improve and sustain healthcare quality. Hospitals searching for the next level of quality management can use this evidence-based framework as a roadmap to translate their vision on quality into daily practice. Testing the implementation and utility of the roadmap in real-world practice settings is a next research priority. We will conduct pilot projects to test, implement and further develop the roadmap and to relate sustainable outcomes such as benefits for patients and healthcare professionals. The first experiences of the roadmap implementation in two pilot projects are positive. Clinicians indicate that reflecting with all stakeholders about quality encourages them to take more ownership. According to new insights and feedback from pilot projects, the roadmap will be revised and further validated. While several exciting opportunities exist for the application and extension of the co-creation roadmap, further international research is needed to fully understand its relevance, transferability and reach in the global context.

## Conclusions

In this paper we propose FlaQuM, a new, unique co-creation roadmap towards sustainable healthcare quality to guide researchers, policymakers, hospital managers and clinicians in the sustainability landscape. This co-creation model, of which the content validity is based on the triangulation of multiple forms of evidence like a narrative review and input from international and national experts, clinicians and hospital managers, suggests that a manageable number of six drivers, 19 building blocks and 104 evidence-based action fields may drive the sustainable incorporation of quality into the daily workflow. By focusing on co-creating quality with patients and all relevant stakeholders, we aim to regain commitment, ownership and engagement to quality as growing concerns about sustainability of current hospital quality policies raised. Therefore, FlaQuM can serve as a roadmap to support future sustainable quality policies in hospitals. Future mixed-methods studies will help to further refine and validate the roadmap and to examine the accuracy, applicability, transferability and impact on sustainability. This ongoing approach will support the continuous search towards excellence in quality.

## Supporting information

S1 Fig Documents collected for document analysis. (PDF)

S1 Table Summary of included papers. (PDF)

S2 Table Drivers, building blocks and evidence-based action fields. (PDF)
