# Supplementary figures and images for: A co-creation roadmap towards sustainable quality of care: A multi-method study
Source: PLoS One. 2022 Jun 30;17(6):e0269364. doi: 10.1371/journal.pone.0269364 (PMC9246114; doi:10.1371/journal.pone.0269364)

**S1 Fig. Documents collected for document analysis.**

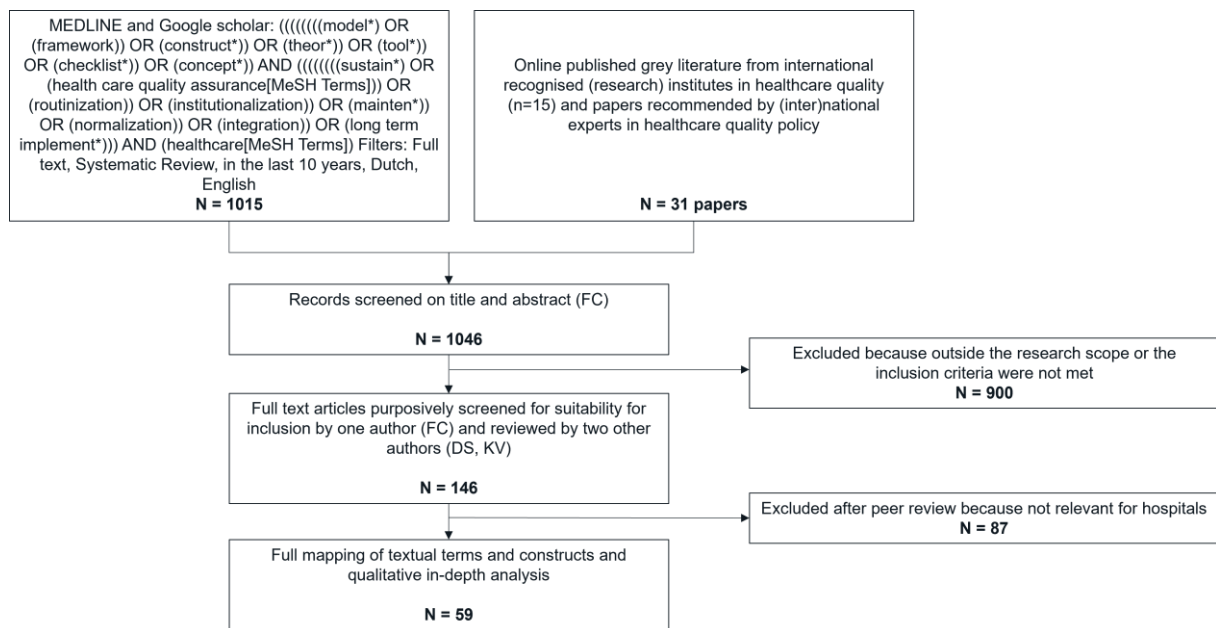

Supplement: S1 Fig — (PDF) [file pone.0269364.s001.pdf]
