# Supplementary material for: A co-creation roadmap towards sustainable quality of care: A multi-method study
Source: PLoS One. 2022 Jun 30;17(6):e0269364. doi: 10.1371/journal.pone.0269364 (PMC9246114; doi:10.1371/journal.pone.0269364)
Supplement: S1 Table — (PDF) [file pone.0269364.s002.pdf]

S2 Table. Summary of included papers.

	**Author**	**Year**	**Country**	**Design**	**Setting**	**Objectives**	**Type of report [Research Article / Grey literature report (Internationally recognised institute in healthcare quality)]**
1	Kabcenell *et al.*	2010	USA and Europe	Observational study in healthcare organisations (n=13) from the US and Europe	Healthcare organisations	To learn if and how healthcare organisations could make dramatic improvements in performance across the organisation, resulting in a considerably more efficient and effective healthcare system.	Grey literature report (Institute for Healthcare Improvement)
2	Kaplan *et al.*	2010	USA	Systematic review (n=47 included articles)	Healthcare organisations and system	1) To identify the contextual factors associated with QI success;<br>2) To categorise, summarise, and synthesise these factors based on their common characteristics and the level of the healthcare system in which they operate;<br>3) To understand the current stage of development of this field of research.	Research Article
3	Maher *et al.*	2010	UK	A co-production approach with front line teams, improvement experts, senior administrative and clinical leaders from within the NHS	Healthcare setting	To support healthcare leaders to implement and sustain effective improvement strategies leading to increased quality and patient experiences at lower cost.	Grey literature report (NHS Institute for Innovation and Improvement)

				and people with specific expertise in the subject area from academia and other industries (number of contributors is not available)			
4	Balik *et al.*	2011	USA	An in-depth review of the research, studied exemplar organisations, and interviewed experts in the field (number of included articles, exemplar organisations or experts is not available)	Hospitals	To identify the primary and secondary drivers of exceptional patient and family inpatient hospital experience (defined as care that is patient-centred, safe, effective, timely, efficient, and equitable), as measured by the Hospital Consumer Assessment of Healthcare Providers and Systems (HCAHPS) survey's "willingness to recommend" the hospital.	Grey literature report (Institute for Healthcare Improvement)
5	O'Leary *et al.*	2012	USA	Literature review (Number of included articles is not available)	Hospitals	To summarise the current understanding of teamwork, describe interventions designed to improve teamwork, and make practical recommendations for hospitals to assess and improve teamwork-related performance.	Research Article
6	Cunningham *et al.*	2012	Australia	Systematic review (n=26 included articles)	Healthcare setting	To conduct a systematic review of studies of professionals' network structures, identifying factors associated with network effectiveness and sustainability, particularly in relation to quality of care and patient safety.	Research Article

7	Lawton *et al.*	2012	UK	Systematic review (n=95 included articles)	Hospitals	To develop a ‘contributory factors framework’ from a synthesis of empirical work which summarises factors contributing to patient safety incidents in hospital settings.	Research Article
8	Meyer *et al.*	2012	USA	Expert opinion of the authors (n=10)	Healthcare setting	To provide guidance for a new, more practical quality measurement policy.	Research Article
9	Wiltsey Stirman *et al.*	2012	USA	Systematic review (n=125 included articles)	Healthcare setting	To review the methods that have been used, the types of outcomes that have been measured and reported, findings from studies that reported long-term implementation outcomes, and factors that have been identified as potential influences on the sustained use of new practices, programs, or interventions	Research Article
10	Healthcare Improvement Scotland	2013	Scotland	A project in partnership with NHS Tayside (Experts were consulted, a literature search and interviews with key informants; number of experts, articles or interviews is not available)	Healthcare organisations	To gain an insight into how ongoing improvement could be embedded into clinical culture to improve the quality of healthcare delivery.	Grey literature report (Healthcare Improvement Scotland)
11	Healthcare Improvement Scotland	2013	Scotland	No comprehensive review of the literature, but the combination of existing resources	Healthcare organisations	1) To increase the understanding of the key issues around spread and sustainability;	Grey literature report (Healthcare Improvement Scotland)

				(secondary studies) in an accessible and practical way (Number of included articles is not available).		2) To signpost readers to existing valuable resources on these topics;<br>3) To assist quality improvement practitioners in the process of planning for spread and sustainability of improvement and its implementation;<br>4) To advise supporting organisations on initiatives that could facilitate spread and sustainability of improvements at a national level.	
12	Lega *et al.*	2013	Italy	Systematic review (n=37 included articles)	Healthcare system and organisations	To present and discuss the streams of knowledge regarding how management can influence the quality and sustainability of health systems and organizations.	Research Article
13	Swensen *et al.*	2013	USA	Based on the results of a 90-Day Innovation Project on leadership, conducted five expert interviews, and convened an expert leaders meeting of 12 recognized organizational leaders	Healthcare organisations	To present three interdependent dimensions of leadership: new mental models, High-Impact Leadership Behaviors, and the IHI High-Impact Leadership Framework.	Grey literature report (Institute for Healthcare Improvement)
14	Groene *et al.*	2014	Europe (Czech Republic, France, Germany, Poland,	Based on state-of-the art research and synthesises the results of the DUQuE Project and other large-scale. empirical studies,	Hospitals	To provide an up-dated framework to assess quality and safety improvement in hospitals.	Grey literature report [‘Deepening our Understanding of Quality Improvement in Europe (DUQuE)’ collaboration]

			Portugal, Spain and Turkey)	systematic reviews, and expert knowledge (number of included articles, empirical studies and experts is not available)			
15	Jeffcott *et al.*	2014	Scotland	Literature review and with face-to-face meetings with subject matter experts (number of included articles and experts is not available)	Healthcare setting	To develop an accessible resource to help healthcare practitioners understand the key factors that impact on the successful spread and sustainability of improvement.	Grey literature report [Healthcare Improvement Scotland (on behalf of NHS Scotland Quality Improvement Hub)]
16	Minnier *et al.*	2014	USA	Based on experiences of the authors in healthcare organisations, such as the University of Pittsburgh Medical Center (number of observed organisations is not available)	Healthcare organisations	To develop an educational program that helps selected staff develop the knowledge and tools required to plan and implement a quality improvement project that will yield lasting results.	Grey literature report (Agency for Healthcare Research and Quality)
17	Agency for Healthcare Research and Quality	2015	USA	Based on the CUSP patient safety model and the experience of the more than 2,000 hospitals that have participated in the CLABSI and CAUTI prevention projects from 2008 through 2015	Hospitals	Not specified.	Grey literature report (Agency for Healthcare Research and Quality)

18	Ament *et al.*	2015	The Netherlands	Systematic review (n=14 included articles)	Healthcare setting	1) To evaluate the state of the art in sustainability research; 2) To evaluate the outcomes of professionals' adherence to guideline recommendations in medical practice.	Research Article
19	de Silva *et al.*	2015	UK	A rapid collation of empirical research n=73 articles about the NHS were analysed, as well as more than 100 studies from other countries as a comparison)	Hospitals and the healthcare system	To compile published research about the key barriers to improvement in the NHS.	Grey literature report (The Health Foundation)
20	Hollnagel *et al.*	2015	Denmark, USA and Australia	Based on expert experiences and based on models used in other settings than the healthcare setting (Number of experts and models are not available)	Healthcare system	To explain the key differences between, and implications of, the two ways of thinking about safety (Safety-I and Safety-II).	Grey literature report (The Resilient Health Care Net)
21	Johnson *et al.*	2015	UK	Systematic review (n=67 included articles)	Healthcare setting	To establish the characteristics of successful behaviour change interventions in healthcare.	Research Article
22	Marimuthu *et al.*	2016	Malaysia	Systematic review (number of included articles not available)	Healthcare setting	To focus on three main conceptual aspects – dimensions of sustainability practices in healthcare, drivers of sustainable practices within the industry and strategies to implement sustainability effectively in healthcare.	Research Article

23	Murray	2015	Australia	Literature review (n=33 included articles)	Hospitals	To explore how community representation in hospital governance is achieved.	Research Article
24	Health Service Executive	2017	Ireland	The Framework is informed by international models and evidence as well as local improvement experience and learning (numbers of models, included articles and case studies not available)	Healthcare organisations	To develop a framework that fluences and guides our thinking, planning and delivery of care in our services	Grey literature report (Health Service Executive)
25	Scoville *et al.*	2016	USA	Literature review and interviews with leading organisations (number of interviews is not available)	Healthcare organisations	To present a framework that healthcare organisations can use to sustain improvements in the safety, effectiveness, and efficiency of patient care.	Grey literature report (Institute for Healthcare Improvement)
26	Willis *et al.*	2016	Canada	Literature review (n=68 included articles)	Healthcare organisations	1) To discuss the guiding principles by which organisational culture change may be sustained in healthcare organisations;<br>2) To discuss the mechanisms by which these principles may operate;<br>3) To discuss the contextual factors that influence the likelihood of these principles being effective.	Research Article
27	Agency for Healthcare	2017	USA and Canada	Observational study of the Agency for	Healthcare organisations	Not specified.	Grey literature report

	Research and Quality			Healthcare Research and Quality (AHRQ) Safety Program for Ambulatory Surgery (in the United States, organisations included Intermountain Healthcare, Virginia Mason Hospital & Medical Center, and ThedaCare; international leaders included Saskatoon Health Region in Saskatchewan Province, Canada).			(Agency for Healthcare Research and Quality)
28	Frankel *et al.*	2017	USA	A group of subject-matter experts at the Institute for Healthcare Improvement (IHI) and Safe & Reliable Healthcare (SRH) that collaborated over 15 years to develop this framework (number of experts is not available)	Healthcare system and organisations	To develop and lay out a practical framework for how any healthcare organisation or system can continuously and reliably improve patient safety.	Grey literature report (Institute for Healthcare Improvement)
29	Gabutti *et al.*	2017	Italy	Systematic review (n=42 included articles)	Hospitals	To make stock of what is known in the field of hospital organisation about how hospitals are changing, as well as of how such change may	Research Article

						be implemented effectively through managerial tools.	
30	Jabbal	2017	England	Based on a roundtable event (n=13), semi-structured interviews with senior NHS leaders (n=5) and stakeholders involved in quality improvement initiatives (n=2), and a literature review (number of included articles is not available).	Healthcare system and organisations	To capture the narratives and practical lessons from leaders of organisations that are already engaged with quality improvement as a routine way of working.	Grey literature report (The King's Fund)
31	Alderwick *et al.*	2017	England	Based on existing literature and examples from within the NHS of where quality has been improved and describing how this was done (number of included articles are not available)	Healthcare system and organisations	Not specified.	Grey literature report (The King's Fund and The Health Foundation)
32	Perlo *et al.*	2017	USA	Based on scans of the current published literature on engagement, satisfaction, and burnout; more than 30 expert interviews based on the literature	Healthcare organisations	1) To serve as a guide for healthcare organisations to engage in a participative process where leaders ask colleagues at all levels of the organization, "What matters to you?" — enabling them to	Grey literature report (Institute for Healthcare Improvement)

				scan, including interviews with patients and exemplar organizations both within and outside of healthcare; site visits; and, finally, learning from 11 health and healthcare systems working to improve joy in work as they participated in a two-month prototype program testing steps, refining the framework, and identifying ideas for improvement		better understand the barriers to joy in work;<br>2) To co-create meaningful, high-leverage strategies to address these issues.	
33	Scoville *et al.*	2017	USA	A pilot test of key sustainability practices in two ambulatory surgery centers in a project sponsored by AHRQ in collaboration with the Health Research & Educational Trust (HRET)	Healthcare organisations	Not specified.	Grey literature report (Institute for Healthcare Improvement)
34	Breyer *et al.*	2019	Brazil	Systematic review (n=35 included articles)	Hospitals	To identify and describe hospital quality indicators, classifying them according to Donabedian's structure, process and outcome model and in specific domains	Research Article

						(quality, safety, infection and mortality) in two care divisions: inpatient and emergency services.	
35	Geerligs *et al.*	2018	Australia	Systematic review (n=43 included articles)	Hospitals	To identify and explore relationships between these barriers and facilitators to highlight key domains that need to be addressed by researchers and clinicians seeking to implement hospital-based, patient-focused interventions.	Research Article
36	Hilton *et al.*	2018	USA	Based on existing research, methods, and examples, and with a focus on Everett Rogers' early adopters and early majority categories (number of included articles, methods and examples is not available)	Healthcare organisations	To present a framework and set of methods for the psychology of change — five interrelated domains of practice that organizations can use to advance and sustain improvement.	Grey literature report (Institute for Healthcare Improvement)
37	Lennox *et al.*	2018	UK	Systematic review (n=62 included articles)	Healthcare setting	1) To identify what approaches are available to assess and influence sustainability in healthcare;<br>2) To describe the different perspectives, applications and constructs within these approaches to guide their future use.	Research Article

38	Mortimer *et al.*	2018	UK	Expert opinion of the authors and a case study	Healthcare system	1) To identify stages in the quality improvement process at which sustainability is usefully considered and make specific suggestions for its inclusion; 2) To set out a simple approach for incorporating sustainability into mainstream quality improvement methodologies.	Research Article (Centre for Sustainable Healthcare)
39	Shelton *et al.*	2018	USA	Literature review (number of included articles is not available)	Public health and the healthcare setting	To understand what factors and processes influence the sustainability of interventions and how to plan proactively for the continuation of evidence-based interventions.	Research Article
40	Slade *et al.*	2018	Australia	A rapid review (n=16 included articles/frameworks)	Health system	To evaluate frameworks for embedding research into routine allied health practice, as the basis for high quality, safe, efficient and consumer-focused care.	Research Article
41	Daley Ullem *et al.*	2018	USA	IHI Lucian Leape Institute's research scan, evaluation of governance education in quality, and more than 50 interviews with governance experts, health system leaders, and trustees	Healthcare organisations	To reduce variation in and clarify trustee responsibilities for quality oversight, and also serve as practical tools for trustees and the health system leaders who support them to govern quality in a way that will deliver better care to patients and communities	Grey literature report (Institute for Healthcare Improvement)
42	Di Vincenzo	2018	Italy	Observational study in 35 hospitals organisations	Hospitals	To study the dynamics of networking behaviours of hospital organisations.	Research Article

43	World Health Organization Service Delivery and Safety Department	2018	Switzerland	Existing WHO tools and resources on quality improvement are collated	Healthcare system and organisations	To support implementation of quality improvement approaches to make health services more effective, safe and people-centred.	Grey literature report (World Health Organization)
44	Côté-Boileau *et al.*	2019	Canada	A scoping review (n=24 included articles)	Healthcare system	1) To consolidate the evidence on the 3S of healthcare innovation to better understand how they work; 2) To consolidate the mechanisms and contextual conditions that enable complex health systems and organisations to increase uptake of innovations.	Research Article
45	Hailemariam *et al.*	2019	USA	Systematic review (n=26 included articles)	Public health community-based organisations	To summarise the existing evidence supporting discrete sustainment strategies for public health EBIs and facilitating and hindering factors of sustainment.	Research Article
46	MacLeod	2019	Ireland	Expert opinion of the author	Healthcare organisations	Not specified.	Grey literature report (International Society for Quality in Health care)
47	Busse *et al.*	2019	Denmark	Available evidence on different quality strategies is summarised and recommendations for their implementation are provided (number of included articles is not available)	Healthcare system	1) To provide an overall conceptual framework for understanding and applying strategies aimed at improving quality of care; 2) To help policymakers to understand concepts of quality and to support them to	Grey literature report (World Health Organization, The Organisation for Economic Co-operation and Development, The European Observatory

						evaluate single strategies and combinations of strategies.	on Health Systems and Policies supports)
48	Patient Safety Learning	2019	UK	Not specified	Healthcare organisations	To describe the actions needed to make the patient-safe future a reality	Grey literature report (Patient Safety Learning)
49	Plessers *et al.*	2019	Belgium	Literature and expert opinion (number of included articles and experts is not available)	Healthcare system and organisations	1) To define the concept of quality indicator;<br>2) To describe how they can be developed in a systematic, evidence-based way.	Grey literature report [Vlaams Instituut voor Kwaliteit van Zorg (Flemish Institute for Quality of Care)]
50	Braithwaite *et al.*	2020	Australia	Systematic review (n=92 included articles)	Healthcare system and organisations	To describe theoretical frameworks, definitions and measures of sustainability, as applied in published evaluations of healthcare improvement programmes and interventions.	Research Article
51	Canadian Patient Safety Institute	2020	Canada	Based on different models, theories and frameworks (number of models, theories and frameworks not available)	Healthcare organisations	To support teams across all healthcare sectors in using a Knowledge Translation and Quality Improvement integrated approach to change that will impact patient safety outcomes.	Grey literature report (Canadian Patient Safety Institute)
52	Cowie *et al.*	2020	Scotland	Systematic review (n=32 included articles)	Hospitals	To identify, appraise and synthesise the barriers and facilitators that influenced the delivery of sustained healthcare interventions in a hospital-based setting.	Research Article
53	Gandhi *et al.*	2020	USA	Expert opinion and case studies (Number of experts and case studies not available)	Healthcare system and organisations	Not specified.	Research Article

54	Lachman *et al.*	2020	Ireland	Expert opinion of the authors	Healthcare system and organisations	To revise the basic quality framework and to redefine quality with the advantage of the experience gained over the past 20 years.	Research Article
55	MacLeod	2020	Ireland	Expert opinion of the author	Healthcare organisations	Not specified.	Grey literature report (International Society for Quality in Health care)
56	National Steering Committee for Patient Safety	2020	USA	The recommendations are built on the substantial body of experience, evidence, and lessons learned that the NSC has gathered (n=27 organisations)	Healthcare organisations	To illuminate the collective insights of the 27 organisations represented on the National Steering Committee for Patient Safety (NSC), united in their efforts to achieve safer care and reduce harm to patients and those who care for them	Grey literature report (Institute for Healthcare Improvement)
57	O'Donovan *et al.*	2020	Ireland	Systematic review (n=36 included articles)	Healthcare organisations	To identify enablers of psychological safety within the literature in order to produce a comprehensive list of factors that enable psychological safety specific to healthcare teams.	Research Article
58	Shah	2020	UK	Expert opinion of the author	Healthcare system and organisations	1) To explore the difference between quality improvement and a quality management system, by defining quality improvement;<br>2) To describe how to best use quality improvement alongside control, assurance, and planning as part of a more	Research Article

						holistic management system focus on quality.	
59	Healthcare Improvement Scotland	2018	Scotland	Literature review, expert interviews (n=22) and discussions with a wide range of stakeholders across Scotland through a mixture of focus groups (n=18 focus groups) and individual meetings (n=32 individuals)	Healthcare system	To describe the key components and functions of a national quality management system that is tailored and relevant to Healthcare Improvement Scotland and its key national partners.	Grey literature report (Healthcare Improvement Scotland)

References for included papers.

Nr	References
1	Kabcenell A, Nolan TW, Martin L a, Gill Y. The pursuing perfection initiative: lessons on transforming health care. IHI Innov Series white paper. Cambridge, Massachusetts: Institute for Healthcare Improvement; 2010. Available from www.IHI.org
2	Kaplan HC, Brady PW, Dritz MC, Hooper DK, Linam WM, Froehle CM, et al. The influence of context on quality improvement success in health care: A systematic review of the literature. Milbank Q 2010;88(4):500–59. doi:10.1111/j.1468-0009.2010.00611.x
3	Maher L, Gustafson PD, Evans A. Sustainability Model and Guide. Inst Innov Improv NHS 2010. Available from: https://www.england.nhs.uk/improvement-hub/publication/sustainability-model-and-guide/
4	Balik B, Conway J, Zipperer L, Watson J. Achieving an Exceptional Patient and Family Experience of Inpatient Hospital Care. IHI Innovation Series white paper. Cambridge, Massachusetts: Institute for Healthcare Improvement; 2011. Available from www.IHI.org
5	O'Leary KJ, Sehgal NL, Terrell G, Williams M V. Interdisciplinary teamwork in hospitals: A review and practical recommendations for improvement. J Hosp Med 2012;7(1):48–54. doi:10.1002/jhm.970
6	Cunningham FC, Ranmuthugala G, Plumb J, Georgiou A, Westbrook JI, Braithwaite J. Health professional networks as a vector for improving healthcare quality and safety: A systematic review. BMJ Qual Saf 2012;21(3):239–49. doi:10.1136/bmjqs-2011-000187
7	Lawton R, McEachan RRC, Giles SJ, Sirriyeh R, Watt IS, Wright J. Development of an evidence-based framework of factors contributing to patient safety incidents in hospital settings: A systematic review. BMJ Qual Saf 2012;21(5):369–80. doi:10.1136/bmjqs-2011-000443
8	Meyer GS, Nelson EC, Pryor DB, James B, Swensen SJ, Kaplan GS, et al. More quality measures versus measuring what matters: A call for balance and parsimony. BMJ Qual Saf 2012;21(11):964–8. doi:10.1136/bmjqs-2012-001081
9	Wiltsey Stirman S, Kimberly J, Cook N, Calloway A, Castro F, Charns M. The sustainability of new programs and innovations: A review of the empirical literature and recommendations for future research. Implement Sci 2012;7(1):17. doi:10.1186/1748-5908-7-17
10	Healthcare Improvement Scotland. Quality Improvement – sustainable in any organisational culture? 2013;(April):1–27. Available from: file:///C:/Users/u0129426/Downloads/Quality-improvement-sustainable-in-any-organisational-culture.pdf
11	Healthcare Improvement Scotland. Guide on spread and sustainability. Chang its Leadersh role Posit Emot. 2013;(July):39. Available from: http://www.qihub.scot.nhs.uk/
12	Lega F, Prenestini A, Spurgeon P. Is management essential to improving the performance and sustainability of health care systems and organizations? A systematic review and a roadmap for future studies. Value Heal 2013;16:S46–51. doi:10.1016/j.jval.2012.10.004
13	Swensen S, Pugh M, McMullan C, Kabcenell A. High-Impact Leadership: Improve Care, Improve the Health of Populations, and Reduce Costs. IHI White Paper. Cambridge, Massachusetts: Institute for Healthcare Improvement; 2013. Available from www.IHI.org
14	Groene O, Kringos D, Sunol R on behalf of the DUQuE Project. Seven ways to improve quality and safety in hospitals. An evidence-based guide. DUQuE Collaboration, 2014, Available from: www.duque.eu
15	Jeffcott S. The spread and sustainability of quality improvement in healthcare. NHSScotland Quality Improvement Hub. 2014;24. Available from: www.qihub.scot.nhs.uk

**16**	Minnier BTE, He FAC, Officer CQ, Medical P, Exchange I, Board E. How To Build Sustainability Into the Innovation Process | AHRQ Innovations Exchange How To Build Sustainability Into the Innovation Process How To Build Sustainability Into the Innovation Process | AHRQ Innovations Exchange. 2014;1–2. Available from: http://www.fammed.usouthal.edu/Scholarly%20Activities/2014%20How%20To%20Build%20Sustainability%20Into%20the%20Innovation%20Process%20_%20AHRQ%20Innovations%20Exchange.pdf
**17**	Agency for Healthcare Research and Quality (AHRQ). A Model for Sustaining and Spreading Safety Interventions: AHRQ Safety Program for Reducing CAUTI in Hospitals. Published Online First: 2015. Available from: https://www.ahrq.gov/sites/default/files/publications/files/sustainability-guide_2.pdf
**18**	Ament SMC, De Groot JJA, Maessen JMC, Dirksen CD, Van Der Weijden T, Kleijnen J. Sustainability of professionals' adherence to clinical practice guidelines in medical care: A systematic review. BMJ Open 2015;5(12). doi:10.1136/bmjopen-2015-008073
**19**	de Silva D. What 's getting in the way? Barriers to improvement in the NHS. 2015;(24):28. Available from: http://www.health.org.uk/publications/what-s-getting-in-the-way/
**20**	Hollnagel E, Wears RL, Braithwaite J. From Safety-I to Safety-II: A White Paper. The Resilient Health Care Net: Published simultaneously by the University of Southern Denmark, University of Florida, USA, and Macquarie University, Australia. 2015. Available from: https://www.england.nhs.uk/signuptosafety/wp-content/uploads/sites/16/2015/10/safety-1-safety-2-whte-papr.pdf
**21**	Johnson MJ, May CR. Promoting professional behaviour change in healthcare: What interventions work, and why? A theory-led overview of systematic reviews. BMJ Open 2015;5(9). doi:10.1136/bmjopen-2015-008592
**22**	Marimuthu M, Paulose H. Emergence of Sustainability Based Approaches in Healthcare: Expanding Research and Practice. Procedia - Soc Behav Sci 2016;224:554–61. doi:10.1016/j.sbspro.2016.05.437
**23**	Murray Z. Community representation in hospital decision making: A literature review. Aust Heal Rev 2015;39(3):323–8. doi:10.1071/AH14016
**24**	Health Service Executive. Framework for Improving Quality in our Health Service. Hse. 2017;1–25. Available from: https://www.hse.ie/eng/about/who/qid/nationalsafetyprogrammes/decontamination/quality-improvement-framework.pdf
**25**	Scoville R, Little K, Rakover J, Luther K MKS. Sustaining Improvement. IHI White Paper. Cambridge, Massachusetts Inst Healthc Improv. 2016;1–33. Available from www.IHI.org
**26**	Willis CD, Saul J, Bevan H, Scheirer MA, Best A, Greenhalgh T, et al. Sustaining organizational culture change in health systems. J Heal Organ Manag 2016;30(1):2–30. doi:10.1108/JHOM-07-2014-0117
**27**	Agency for Healthcare Research and Quality (AHRQ). A Frontline Management System To Sustain Improvement in Safety Practices. Content last reviewed June 2017. AHRQ, Rockville, MD. Available from: https://www.ahrq.gov/hai/tools/ambulatory-surgery/sections/sustainability/management/kit.html
**28**	Frankel A, Haraden C, Federico F, et al. Frankel A, Haraden C, Federico F, Lenoci-Edwards J. A Framework for Safe, Reliable, and Effective Care. White Paper. Cambridge, MA: Institute for Healthcare Improvement and Safe & Reliable Healthcare; 2017. Available from www.IHI.org
**29**	Gabutti I, Mascia D, Cicchetti A. Exploring "patient-centered" hospitals: a systematic review to understand change. BMC Health Serv Res 2017;17(1):1–16. doi:10.1186/s12913-017-2306-0
**30**	Jabbal J. Embedding a culture of quality improvement. Kings Fund 2017;35. Available from: https://www.kingsfund.org.uk/sites/default/files/2017-11/Embedding-culture-QI-Kings-Fund-November-2017.pdf

**31**	Alderwick H, Charles A, Jones B., Warburton W. Making the case for quality improvement: lessons for NHS boards and leaders. King's Fund, The health Foundation. 2017; (October). Available from: https://www.kingsfund.org.uk/publications/making-case-quality-improvement
**32**	Perlo J, Balik B, Swensen S, Kabcenell A. IHI Framework for Improving Joy in Work. IHI White Paper. Cambridge, Massachusetts: Institute for Healthcare Improvement; 2017. Available from www.IHI.org
**33**	Scoville R. 6 Essentials for sustainable improvement. Institute for Healthcare Improvement; 2017. Available from: http://www.ihi.org/communities/blogs/six-essential-practices-for-sustainable-improvement
**34**	Breyer JZ, Giacomazzi J, Kuhmmer R, Lima KM, Hammes LS, Ribeiro RA, et al. Hospital quality indicators: a systematic review. Int J Health Care Qual Assur 2019;32(2):474–87. doi:10.1108/IJHCQA-04-2018-0091
**35**	Geerligs L, Rankin NM, Shepherd HL, Butow P. Hospital-based interventions: A systematic review of staff-reported barriers and facilitators to implementation processes. Implement Sci 2018;13(1):1–17. doi:10.1186/s13012-018-0726-9
**36**	Hilton K, Anderson A. IHI Psychology of Change Framework to Advance and Sustain Improvement. IHI White Paper. Boston, Massachusetts: Institute for Healthcare Improvement; 2018. Available from www.IHI.org
**37**	Lennox L, Maher L, Reed J. Navigating the sustainability landscape: A systematic review of sustainability approaches in healthcare. Implement Sci 2018;13(1):1–17. doi:10.1186/s13012-017-0707-4
**38**	Mortimer F, Isherwood J, Wilkinson A, Vaux E. Sustainability in quality improvement: redefining value. Futur Healthc J 2018;5(2):88–93. doi:10.7861/futurehosp.5-2-88
**39**	Shelton RC, Rhoades Cooper B, Stirman SW. The Sustainability of Evidence-Based Interventions and Practices in Public Health and Health Care. Annu Rev Public Heal 2018;39:55–76. doi:10.1146/annurev-publhealth
**40**	Slade SC, Philip K, Morris ME. Frameworks for embedding a research culture in allied health practice: A rapid review. Heal Res Policy Syst 2018;16(1):1–15. doi:10.1186/s12961-018-0304-2
**41**	Daley Ullem, E. Ganhdi T, Mate K, Whittington, J,. Renton M, Joellen H. Framework for Effective Board Governance of Health System Quality. IHI White Paper. Boston, Massachusetts: Institute for Healthcare Improvement; 2018. Available from www.IHI.org
**42**	Di Vincenzo F. Exploring the networking behaviors of hospital organizations. BMC Health Serv Res 2018;18(1):1–10. doi:10.1186/s12913-018-3144-4
**43**	Improving the quality of health services: tools and resources. Turning recommendations into practice. Geneva: World Health Organization; 2018. Available from: https://www.who.int/publications/i/item/9789241515085
**44**	Côté-Boileau É, Denis J-L, Callery B, Sabean M. The unpredictable journeys of spreading, sustaining and scaling healthcare innovations: a scoping review. Health Research Policy and Systems 2019;17(84):1-26. doi:10.1186/s12961-019-0482-6
**45**	Hailemariam M, Bustos T, Montgomery B, Barajas R, Evans LB, Drahota A. Evidence-based intervention sustainability strategies: A systematic review. Implement Sci 2019;14(1):1–12. doi:10.1186/s13012-019-0910-6
**46**	MacLeod, H. 3 pillars of sustainable quality and patient safety improvement. International Society for Quality in Health Care. 2019. Available from: https://isqua.org/latest-blog/3-pillars-of-sustainable-quality-and-patient-safety-improvement.html
**47**	Busse R, Klazinga N, Panteli D, Quentin W. Health Policy Series No. 53 The editors Improving healthcare quality in Europe Characteristics, effectiveness and implementation of different strategies. 2019. Available from: www.healthobservatory.eu

**48**	Patient Safety Learning. Patient safety learning The Patient-Safe Future: A Blueprint for Action. 2019;1–98. Available from: www.patientsafetylearning.org
**49**	Plessers M, Ghekiere A, De Wachter D, Deneckere S, Tambuyzer E, Ramaekers D. Het ontwikkelen van evidence-based indicatoren van kwaliteit van zorg in Vlaanderen: een methodologie. Vlaams Inst voor Kwal van Zorg vzw 2019;:1–13.
**50**	Braithwaite J, Ludlow K, Testa L, Herkes J, Augustsson H, Lamprell G, et al. Built to last? The sustainability of healthcare system improvements, programmes and interventions: a systematic integrative review. BMJ Open 2020;10(6):e036453. doi:10.1136/bmjopen-2019-036453
**51**	Canadian Patient Safety Institute. A Guide to Patient Safety Improvement: Integrating Knowledge Translation & Quality Improvement Approaches. Edmonton, Alberta. 2020. Available from: www.patientsafetyinstitute.ca
**52**	Cowie J, Nicoll A, Dimova ED, Campbell P, Duncan EA. The barriers and facilitators influencing the sustainability of hospital-based interventions: A systematic review. BMC Health Serv Res 2020;20(1):1–27. doi:10.1186/s12913-020-05434-9
**53**	Gandhi TK, Feeley D, Schummers D. Zero Harm in Health Care. NEJM Catal 2020;1(2):1–23. doi:10.1056/cat.19.1137
**54**	Lachman P, Batalden P, Vanhaecht K. A multidimensional quality model: an opportunity for patients, their kin, healthcare providers and professionals to coproduce health. F1000Research 2020;9:1140. doi:10.12688/f1000research.26368.2
**55**	MacLeod, H. Sustainable Quality Improvement Emotion, Inspiration, and Creativity. International Society for Quality in Health Care. 2020. Available from: https://isqua.org/latest-blog/sustainable-quality-improvement-emotion,-inspiration,-and-creativity.html
**56**	National Steering Committee for Patient Safety. Safer Together: A National Action Plan to Advance Patient Safety. Boston, Massachusetts: Institute for Healthcare Improvement; 2020. Available from www.ihi.org/SafetyActionPlan
**57**	O'Donovan R, McAuliffe E. A systematic review of factors that enable psychological safety in healthcare teams. Int J Qual Heal Care 2020;32(4):240–50. doi:10.1093/intqhc/mzaa025
**58**	Shah A. How to move beyond quality improvement projects. BMJ 2020;370:1–5. doi:10.1136/bmj.m2319
**59**	Healthcare Improvement Scotland. Quality Management System: A 90-day innovation cycle. 2018;1–24. Available from: http://www.healthcareimprovementscotland.org/previous_resources/policy_and_strategy/quality_management_system.aspx
