# Supplementary material for: A co-creation roadmap towards sustainable quality of care: A multi-method study
Source: PLoS One. 2022 Jun 30;17(6):e0269364. doi: 10.1371/journal.pone.0269364 (PMC9246114; doi:10.1371/journal.pone.0269364)
Supplement: S2 Table — (PDF) [file pone.0269364.s003.pdf]

S3 Table. Drivers, building blocks and evidence-based action fields.

Six drivers	19 building blocks	Evidence-based action fields (not exhaustive)
**1. Quality Design and Planning**	1.1 Define a shared vision, set the aims, prioritise and focus	- A shared vision on quality is defined from a multidimensional perspective and this vision is reflected in everything the organisation does, including technical dimensions and core values [4, 25, 26, 30, 31, 33, 37, 48, 54, 58].<br>- Patients, their kin as well as providers are central in the (established) aims [4, 24, 25, 28-30, 32, 53, 54, 56].<br>- Quality is prioritised as a key strategic goal of the organisation [30, 38, 48, 53].<br>- Resources to sustain quality are allocated [26, 35, 37, 38, 45, 51].<br>- The focus is on progress by small steps, giant leaps forward are not expected [26, 30, 31, 46].
	1.2 Involvement of stakeholders	- The organisation has a 'people matter'-mindset: buy-in of all stakeholders at all levels is ensured to codesign the quality vision and aims [1, 14, 15, 25, 23, 30-32, 36, 37, 40, 47, 53, 55].<br>- The stakeholders for the whole quality management model are mapped from the inception towards sustainability [3, 17, 26, 35, 44-46, 51, 59].<br>- Their perspectives, experiences, interests and needs are understood to create value for all stakeholders [16, 25, 30, 32, 35, 39, 44, 52, 53, 59].<br>- Competing demands and interests are made transparent [22, 25, 31, 33, 58].<br>- Competing demands are negotiated and navigated to gain genuine agreement on value creation [9, 15, 28, 30, 37, 44].
	1.3 Adaptability and fit	- The quality vision and aims fit with internal and external demands and priorities [11, 25, 26, 37].<br>- The vision and aims are adapted in the language, culture and structure of the organisation [3, 9, 16, 19, 25, 35, 37, 52].<br>- Adaptability and fit are balanced by identifying and understanding local barriers [3, 11, 17, 21, 39, 44, 45, 51, 52].<br>- The known change methods and models are identified in the organisation so innovations can be adapted and adopted smoothly [10, 15, 25, 28, 30, 46, 48, 51, 52, 58].
**2. Quality control**	2.1 Legal and technical requirements and audits	- An up-to-date overview of legal requirements for audit or inspection of clinical services, supporting departments and technical facilities is available [7, 14, 35, 40, 44, 47, 48, 52, 56, 58].<br>- An up-to-date overview of the specifications for voluntary audits and labels is available [7, 14, 25, 30, 35, 47, 52, 58].<br>- There is a coordinating body and contact person per clinical/technical/organisational department for the quality control and follow-up [26, 35, 48].

		- The internal and external reporting lines (e.g. annual reports) and deadlines are defined as well as planned based on the standards for both legal requirements as well as voluntary label achievements [8, 14, 28].
	2.2 Monitoring system	- Indicators and indicator specific targets are defined based on the vision and aims of the organisation [15, 25, 26, 31, 51].<br>- A mix between structure, process, outcome and balancing indicators and a balance between soft and hard metrics are the focus [26, 30, 37, 44, 46, 48, 51, 52, 58].<br>- For each indicator the monitoring system (automated controls or controls by the workforce) and the level of data collection (data on individual or aggregated level) is defined [2, 8, 11, 31, 34, 49].<br>- The monitoring system to ensure benchmarking, focus on variation and longitudinal follow-up is defined [3, 25, 28, 48].<br>- Investments are made in the required human, IT and financial resources for a quality monitoring system [2, 8, 9, 14, 17, 22, 24, 30].<br>- There is an open and clear communication to motivate staff to take on challenges and to feel safe about reporting quality issues [15, 25, 28, 35, 48].<br>- The quality monitoring system is an ongoing process [21, 25, 43, 50, 52, 56].
	2.3 Transparent feedback system	- The monitored measurements are communicated real-time, with continuous feedback loops, benchmarked where possible, longitudinal monitoring and variation within and between organisations is made transparent [9, 14, 21, 30-32, 37, 44, 47, 50].<br>- The target audience understands the data source, the data collection method, the data analysis and the visualisation of the data to enhance the credibility and ownership [15, 31, 53].<br>- The level of detail of the data (aggregated or individual) with the target audience made transparent (individual patients and their kin, individual providers, teams, departments, management, board, partner organisations, patient advocacy groups, government or society) is defined [11, 13, 27, 28, 53].<br>- Visual management is applied with actionable data or run charts on learning boards so the target audience is able to use and interact with real-time data to drive improvement [15, 17, 27, 25, 28, 33, 43, 53, 58].<br>- Feedback on positive outcomes and achievements is included in the feedback system [17, 20, 32, 36, 52, 53].
	2.4 Demonstrate the evolution over time on effectiveness and	- The quality monitoring system is able to focus on trends [3, 9, 16, 28, 37, 52, 58].<br>- The link between the monitored data and the improvement intervention is clear [3, 15, 31, 37, 45, 48, 51, 52].

	prioritise new challenges	- An overview of quality improvement indicators and initiatives and follow-up of quality improvement projects is accurate and available for all staff [1, 8, 28, 33, 49, 56].<br>- Quality initiatives are prioritised based on trends of the monitoring system, the advice of internal and external inspections or evidence [21, 24, 25, 28, 40, 51].<br>- The value of the improvement projects (cost/benefit) is made transparent [8, 13, 15, 22, 32, 38, 58].
**3. Quality Improvement**	3.1 Evidence based interventions	- An intervention is developed based on the findings of the quality monitoring system [24, 34, 39, 49].<br>- The intervention is defined based on the analysis of the current process, by identifying the characteristics and causes of poor quality [15, 25, 30, 31, 48].<br>- The content of the (bundled) intervention is based on the latest evidence and knowledge is created by combining research, practice and the experiences of patients, their kin and staff [3, 4, 9, 14-16, 18, 19, 21, 24, 28, 35, 40, 45, 48, 51, 52, 58].<br>- All staff members have access to and knowledge of the latest evidence [15, 17, 35, 40, 46, 48].
	3.2 Teamwork	- All quality improvement projects are performed by multidisciplinary teams and individuals with different skills, experiences, knowledge and viewpoints [2, 5, 17, 35, 52].<br>- Teams work on their relationship by enhancing shared goals, shared knowledge and mutual respect and communicate frequent, timely, accurate and problem-solving [7, 5, 26-28, 31, 33, 35, 37, 46, 51, 55, 58].<br>- Collaboration between and within teams occurs within a higher teamwork climate: a focus exists on positive, trusting relationships, psychological safety and familiarity [5, 26, 32, 44, 55, 57].<br>- Management support activities to enhance joy in work [30, 32, 45].<br>- Teams continuously improve their collaboration and implementation strategies by reflecting back and planning forward [25, 28, 32, 44].
	3.3 Intervention implementation by adapting quality design	- An overall consistent approach is defined for quality improvement, implementation and sustainability [24, 25, 30-33].<br>- State of the art implementation methods and strategies are chosen with respect to micro, meso and macro culture and context. [2, 9, 11, 14, 26, 51].<br>- The content of the intervention and the implementation methods are discussed with the involved teams to enhance the sustainability of current improvement and future new designs [32, 35, 43, 51].<br>- Redesign is performed with respect to human factors (and systems engineering) [1, 7, 15, 28, 36, 48].<br>- Processes and procedures are only standardized where possible and necessary [7, 14, 25, 28, 33, 54, 58].<br>- Processes and procedures are translated and integrated so they encompass the chosen change and methods used so teams understand the 'what, why and how' of the new design [7, 14, 25, 28, 33, 54, 58].

	3.4 Communication and reflection	- There is clear communication about the why of the redesign, the content of the new design and the change methodology [11, 15, 30, 35, 39, 44, 46].<br>- Staff is motivated to critically reflect on the redesign and new design process [16, 20, 28, 33, 36, 52, 58].<br>- The team reflects together to understand the relation between the intervention, the implementation method and the outcomes for patients, their kin and providers [28, 44, 51].<br>- Successful change projects and positive trends in indicators are celebrated and communicated organisation-wide, to patients and their kin and to the community [3, 4, 17, 26, 30, 32, 35, 36, 48, 53, 58].<br>- New knowledge about quality improvement, implementation and sustainability is shared by team members themselves across different teams and partner organisations, In doing so, the leadership role of individual team members is strengthened. [11, 15, 26, 28, 31, 48, 52, 53, 58].
**4. Quality Leadership**	4.1 Personal and clinical leadership	- Every healthcare provider is a clinical leader: he is purposeful, committed, understands the needs of patients, their kin and colleagues, participates in codesign initiatives, inspires and thinks critically [1, 2, 5, 9, 15, 25, 26, 28, 31, 32, 36, 47, 48, 53, 59].<br>- Clinical leaders actively support the organisational goals (quality design, quality control, quality improvement) and facilitate all team members to contribute their views, expertise, and ideas [3, 5, 10, 13, 15-17, 21, 25, 26, 32, 36, 57, 58].<br>- Leaders show integrity and lead by example in actions and language [4, 10, 13, 15, 32, 55, 57].<br>- Leaders create a healthy environment for staff, patients and their kin with psychological and physical safety, trust, value alignment and respect for everyone [15, 22, 28, 32, 48, 53, 55-57].<br>- Clinical leaders feel safe to share their expertise and experiences with all other staff members from bedroom to boardroom [12, 22, 32].<br>- Leadership development is supported by the management [12, 13, 24, 25].
	4.2 Visible, supportive management and staff members	- Management is visible and accessible to the frontline teams so that managers understand the complex operational challenges, bottlenecks and barriers in order to improve those in practice [13, 15, 16, 25, 27, 30, 48, 52].<br>- Ongoing support is ensured by motivating frontline teams and enabling them to engage in quality and to continue to improve and learn [9, 12, 16, 24- 28, 30, 31, 35].<br>- Managers take 'a systems view': they coach, facilitate, coordinate and actively participate in projects with staff in order to build staff capacity and expertise for quality [14, 25, 30, 32, 33, 38, 48, 53].<br>- Strong quality champions advocate, adapt and embed the use and importance of quality into their daily routine [2, 13, 15- 17, 21, 35, 48, 51, 52, 57].

		- Management and staff members are aware of the new evidence or change methods on quality design, quality control and quality improvement and share their own knowledge with external partners [14, 15, 28, 32, 36, 40, 45].<br>- Management show dignity, respect and their appreciation for the commitment of all stakeholders [4, 17, 30, 32, 58].
	4.3 Executive and governance support	- Executives and boards ensure that quality is a strategic priority in the organisation that informs every action and is the centre of all we do [1, 4, 13, 23, 24, 30-33, 45, 48, 53, 56, 58].<br>- Within the board are specific board members have expertise and experience in quality [12, 24, 28, 30, 31, 41, 58].<br>- Executives and boards set quality on the policy agenda to discuss quality indicators as well as individual stories during meetings [1, 14, 31, 36, 48, 53].<br>- Executives support, encourage and enable engagement of management, staff and patients and their kin in quality design, quality control and quality improvement [1, 3, 30, 31, 53, 58].<br>- Executives are visible and accessible to the entire organisation from boardroom to bedroom [24, 30, 32, 47, 57].<br>- Involvement and commitment to quality improvement is demonstrated by the executives and boards [1, 14, 25, 30, 31, 41, 43-46, 48, 58].<br>- Board members actively contribute to the quality management structure and the communication lines are clear [30, 31, 33, 48, 57].
**5. Quality Culture**	5.1 Attitudes and commitment	- Everybody lives up to the core values of quality: dignity and respect, partnership and co-production, holistic care and kindness with compassion [3, 4, 14, 30, 32, 36, 48, 54, 56].<br>- All staff, patients and their kin are motivated, engaged, ready for change and believe in the aims of the quality design, the quality control and the quality improvement of the organisation [1, 9, 14-17, 24, 26, 30, 32, 35-37, 51- 53, 55, 56].<br>- When there are quality problems or patient safety incidents, all staff approach each other respectfully [28, 32, 48, 57].<br>- All staff take ownership and accountability for the relation with every patient and their kin, every colleague and the organisation [27, 28, 35, 43, 48, 52, 57, 58].<br>- Everybody participates in celebrating successes which are internally and externally communicated [17, 30, 32, 53].<br>- Everybody is aware that healthcare is a complex and high-risk environment where individual actions and organisational systems act together [17, 28, 48].

	5.2 Just Culture	- A just culture is supported by balancing between accountability and support at every level of the organisation [10, 27, 28, 57, 53].<br>- Initiatives to enhance psychological safety of patients, their kin and providers are supported throughout the organisation, both within and between all departments, professions and stakeholders [28, 32, 48, 53, 56, 57].<br>- All staff, patients and their kin experience a blame free environment with trust, inclusion, dignity and respect [10, 25, 30, 32, 48, 53, 56, 57].<br>- Individuals are encouraged and feel safe to report errors or near misses and all type of quality concerns and seek solutions to problems without fear, negative consequences or obstacles for learning [7, 17, 28, 32, 35, 48, 57].
	5.3 Continuous learning and innovation	- This organisation is a learning organisation with an embedded quality culture and the 'science of improvement' [17, 43, 46, 48, 51, 53, 57, 58].<br>- The safety-I and safety-II principles are used throughout the organisation and known to all stakeholders [1, 20, 48, 53].<br>- All staff get the opportunity to learn from positive and negative outcomes [1, 17, 28, 53, 57].<br>- All staff are engaged in problem solving and feel safe to suggest improvement actions, projects and movements [28, 39, 48].<br>- Quality innovations are performed proactively rather than reactively [28, 32, 48, 53, 54, 56].<br>- The generated knowledge from innovation is shared with internal and external partners [8, 48, 56, 58].<br>- While continuously learning and innovating, all internal and external bottlenecks, challenges and opportunities are explored [1, 3, 28, 48].
**6. Quality Context**	6.1 Organisational characteristics	- There are financial resources available to facilitate quality design, quality control, quality improvement, quality leadership and quality culture [2, 16, 17, 35, 37, 40, 45, 52].<br>- There is an unambiguous structure of quality from bedroom to boardroom, with defined roles, job descriptions and communication requirements [3, 7, 10, 14, 23, 25, 26, 30, 33, 35, 40, 43, 46, 48, 52].<br>- The organisation invests in appropriate staff levels and protected time to enhance and sustain quality. A competency framework is available to track and ensure that all staff are qualified to perform their work and be accountable [14, 25, 30, 35, 40, 48, 52].<br>- A capacity and capability building system is in place with training and education for all staff to monitor the organisational readiness for change [1, 7, 9, 15-17, 23, 30, 31, 35, 37-39, 45, 47, 48, 51-53, 58].<br>- The appropriate technical resources for quality control, quality improvement and quality sustainability are in place [7, 19, 29, 52, 58].

		- Individuals, teams and departments work in a standardised and uniform way across the organisation [25, 27, 28, 33, 48].<br>- The organisation collaborates with external partners with respect to their policy, system and culture [6, 13, 30, 31, 34, 40, 42, 52].
	6.2 Healthcare system and external policy and demands	- The legislation, ethical and governmental policies are clear and support the organisations' internal and cross boundary collaborations on quality [2, 30, 35, 40, 44, 48, 52].<br>- The healthcare system provides financial incentives to improve and sustain quality [7, 9, 26, 47].<br>- The role of external governmental and non-governmental bodies in quality design, quality control and quality improvement are transparent and unambiguous [14, 47, 56].<br>- A synergy exists between the timeframe and local quality improvement approach on the one hand and the national approach to measure and monitor performance on the other hand [14, 23, 28, 30, 34, 44].<br>- The quality vision and aims, timing and methods used by external partners are in line with the organisation specific quality design, quality control and quality improvement [14, 26, 30, 35, 39, 44].<br>- External societal demands are in line with the internal vision on the multidimensional perspective of quality [22, 23, 29, 30, 32, 38, 53, 56].

REFERENCES

See 'S2 Table. Summary of included papers'.
